# The effect of duration of untreated psychosis and treatment delay on the outcomes of prolonged early intervention in psychotic disorders

**DOI:** 10.1038/s41537-017-0034-4

**Published:** 2017-09-26

**Authors:** Nikolai Albert, Marianne Melau, Heidi Jensen, Lene Halling Hastrup, Carsten Hjorthøj, Merete Nordentoft

**Affiliations:** 1 Copenhagen Mental Health Centre, Capital Region, Copenhagen, Denmark; 2 University of Copenhagen, Copenhagen, Denmark; 3 Psychiatric Research Unit, Region Zealand, Slagelse, Denmark

## Abstract

The duration of untreated psychosis (DUP) has been shown to have an effect on outcome after first-episode psychosis. The premise of specialized early intervention (SEI) services is that intervention in the early years of illness can affect long-term outcomes. In this study, we investigate whether DUP affects treatment response after 5 years of SEI treatment compared to 2 years of SEI treatment. As part of a randomized controlled trial testing the effect of prolonged SEI treatment 400 participants diagnosed within the schizophrenia spectrum were recruited. For this specific study participants were dichotomized based on DUP, treatment delay, and time from first symptom until start of SEI treatment. The groups were analyzed with regard to treatment response on psychopathology, level of functioning, and cognitive functioning. The participants with a short DUP had a tendency to respond better to the prolonged treatment with regards to disorganized and negative dimension. For participants with short duration from first symptom until start of SEI treatment there was a significant difference on the negative dimension favoring the prolonged OPUS treatment. The finding of an effect of prolonged treatment for participants with a short total treatment delay could mean that prolonged SEI treatment is more beneficial than treatment as usual (TAU) so long as it is provided in the early years of illness and not just in the early years after diagnosis.

## Treatment timing: The earlier the better

The duration of untreated psychosis influences the long-term outcomes of treatment. Nikolai Albert, at the Copenhagen Mental Health Centre, and a team of Danish researchers have investigated the effects of a specialized early intervention program (OPUS) in 400 patients diagnosed with schizophrenia spectrum disorders and compared the effects of OPUS after two and five years. Their findings suggest that five years of specialized early intervention was most beneficial when the total duration from symptom start to treatment was shorter than 6 months. The treatment was particularly effective at improving patients’ disorganized behavior and negative symptoms such as blunted emotions and lack of motivation. These findings support previous studies suggesting that patients are more responsive to treatment in the early years of illness and highlight the importance of avoiding delays within the mental health service provision.

## Introduction

The critical phase theory from which early intervention in the psychosis field grew was proposed in the early to mid-nineties.^1, 2^ Based on studies of patients with first-episode schizophrenia it appeared that the first 5 years after onset of illness were more fluctuating compared to the later years, when symptom level and functional deficits seemed to stabilize.^3, 4^ Based on this background, it was suggested that interventions in the early years could have a disproportionally large impact on the long-term outcome of the illness than interventions in the more static later years. Randomized clinical trials have confirmed that psycho-social treatment programs with assertive treatment, social-skill training, and family involvement can exert an impact on the early course of first-episode psychosis.^5^ The effects seen on long-term outcomes have been more contradictory.^6–8^ The specialized early intervention (SEI) programs that were established in many developed countries typically treat patients from 1 to 3 years after their initial diagnosis, not for the entire first 5 years as originally suggested.

Even if the intent of the SEI treatment is to treat patients as early as possible after onset of illness, implementation of SEI services has not uniformly led to a reduction in the time from onset until treatment start.^9^ Subsequently, another part of the early intervention movement focused on the impact of duration of untreated psychosis (DUP) and initiatives to reduce the time from onset of illness to initiation of treatment.^9^ The DUP has been found in several studies and meta-analyses to have an impact on clinical and functional outcomes.^10–12^ Whether DUP is a marker or determinant of outcome has been debated^13, 14^ and several predictors of outcome have been found to be associated with DUP.^12^ One of these is mode of onset; it has been argued that DUP is a marker of onset mode rather than an independent predictor of outcome.^15, 16^ It is still assumed that DUP is one of the few malleable predictors of outcome and, therefore, should be a focus for intervention.^10^ One large randomized clinical trial, testing the effect of society-level awareness campaigns to reduce the DUP, showed that the campaigns significantly reduced the DUP and thereby positively affect the functional level 10 years later.^17^

The two directions of the early intervention field have often failed to integrate^18^ leading to patients in areas with a DUP program being treated in community health centers after identification and treatment facilities targeting first-episode psychosis still treating patients with a long DUP. If the aim of the SEI treatment is to provide high-resource treatment as early as possible after first symptoms and at least within the first 5 years of illness, it is imperative that the DUP not be so long as to place the treatment outside of the critical phase.

Using data from a study of prolonged SEI treatment with no integrated program to shorten DUP we explored (a) whether DUP influenced treatment response, (b) whether delays within the mental health systems exerted an influence on response to the SEI treatment, (c) whether mode of onset was associated with DUP, and (d) whether mode of onset could explain the effect of DUP on outcome.

## Results

### Baseline values

A total of 317 participants had a psychotic diagnosis within the schizophrenia spectrum. Of these 296 had completed the DUP assessment. The mean age at baseline was 25.1 years (SD 4.1) and 49.7% were women. 94.6% had a diagnosis of schizophrenia. The mean DUP was 142 weeks (SD 186, median = 52, range = 962) and the mean treatment delay from register diagnosis of schizophrenia until start of OPUS treatment was 38 weeks (SD 71, median = 14, range = 440). Psychopathological-, functional-, and cognitive-scores for the groups are depicted in Table 1. As shown in Table 1 there was no significant effect of DUP, treatment delay or total treatment delay on the psychopathological-, functional-, or cognitive-scores at baseline of the study (19 months after initiation of treatment). There was a tendency for the functional level to be higher in participants with a short DUP and short total treatment delay.

**Table 1:** Baseline values (on average 19 months after initiation of specialized treatment)

	DUP in months				Treatment delay in months				Total treatment delay in months			
	≤3	>3	Estimated mean difference	*p*	≤3	>3	Estimated mean difference	*p*	≤6	>6	Estimated mean difference	*p*
Psychotic dimension, mean (SD)	1.9 (1.2)	2.2 (1.2)	0.23 (−0.09; 0.55)	0.16	2.1 (1.2)	2.1 (1.3)	0.06 (−0.33;0.21)	0.67	1.9 (1.4)	2.1 (1.2)	0.23 (−0.14; 0.60)	0.22
Disorganized dimension, mean (SD)	0.46 (0.53)	0.50 (0.64)	0.04 (−0.12; 0.2)	0.62	0.48 (0.57)	0.52 (0.66)	0.04 (−0.10;0.18)	0.55	0.43 (0.51)	0.50 (0.63)	0.07 (−0.11;0.25)	0.45
Negative dimension, mean (SD)	1.8 (0.81)	2.0 (1.0)	0.21 (−0.04; 0.46)	0.10	2.1 (0.98)	1.9 (0.95)	−0.12 (−0.33; 0.09)	0.27	1.8 (0.91)	2.0 (0.96)	0.20 (−0.08; 0.48)	0.17
Total PSP, mean (SD)	49.5 (11.8)	46.7 (12.5)	−2.8 (−6.0;0.35)	0.08	47.7 (12.0)	46.2 (12.9)	−1.4 (−4.2; 1.3)	0.30	50.4 (11.8)	46.8 (12.4)	−3.6 (−7.2; 0.10)	0.06
Total BACS *z*-scores, mean (SD)	−2.5 (1.8)	−2.6 (1.8)	−0.12 (−0.59; 0.34)	0.61	−2.7 (1.9)	−2.6 (1.7)	0.13 (−0.28; 0.53)	0.55	−2.7 (1.9)	−2.6 (1.7)	0.15 (−0.39; 0.68)	0.59

Table shows values for participants with duration of untreated psychosis (*DUP*) and treatment delay of ≤ or >3 months and total treatment delay (DUP + treatment delay) of ≤ or >6 months

*PSP* Personal and Social Performance scale, *BACS* Brief Assessment of Cognition in Schizophrenia

### Attrition

**Fig. 1:**
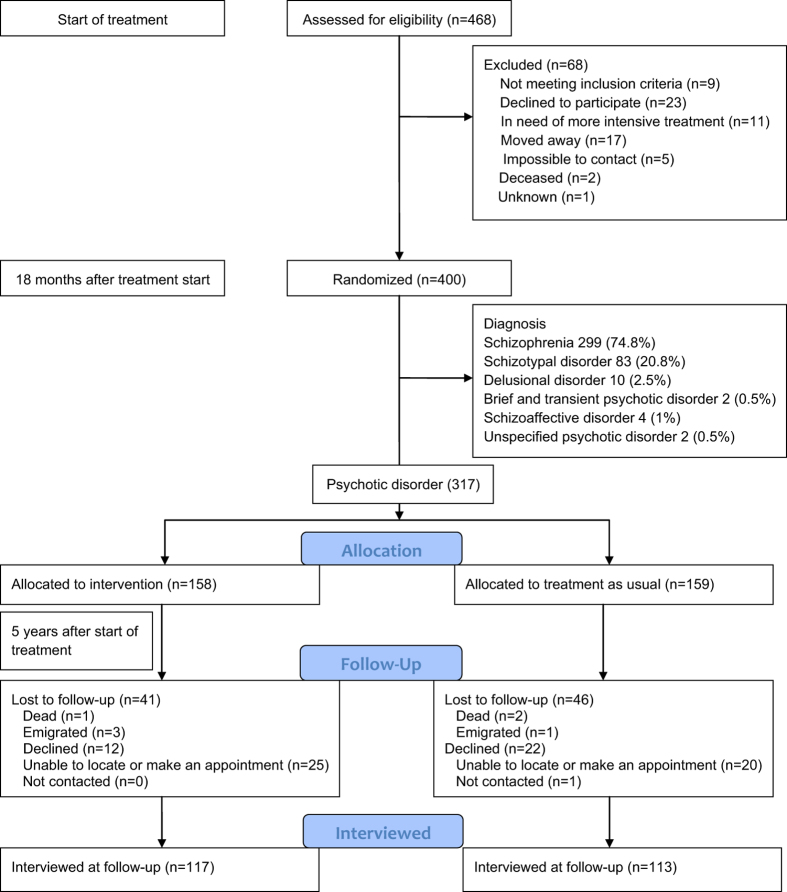
Flow chart, depicting recruitment, and flow of participants

A total of 117 (74%) of the participants randomized to prolonged OPUS treatment and 113 (71%) of the participants randomized to treatment as usual (TAU) attended the follow-up assessment. Attrition analyses showed a significant bias with those with higher scores on the psychotic and disorganized dimensions being less likely to attend follow-up. There was no attrition bias with regard to treatment allocation or DUP. Using multiple imputations we included all participants and corrected for attrition bias. Participant flow is depicted in Fig. 1. Eleven participants were diagnosed with a psychotic schizophrenia spectrum disorder prior to their 18th birthday and were, therefore, initially treated in the adolescent mental health services; exclusion of these participants from the analyses did not change the overall results.

### Outcomes

#### Duration of untreated psychosis

In this study 79 participants had a DUP less than 3 months; 34 of those were randomized to TAU and 45 to prolonged OPUS treatment. As shown in Table 2, there was a tendency for participants with a short DUP to respond better to the prolonged OPUS treatment compared to TAU, with regards to disorganized dimension (estimated mean difference −0.39, 95% confidence interval ((CI) −0.81; 0.03) *p* = 0.07) and negative dimension (estimated mean difference −0.45, 95% CI (−0.96; 0.05)*p* = 0.08). For participants with a long DUP there were no differences between those treated in prolonged OPUS or TAU on any of the outcomes. The interaction analyses indicated a trend for DUP and prolonged treatment to affect negative symptoms (*p* = 0.09). If the short DUP was increased to 6 months the interaction analyses showed a loss of significance. When analyses were conducted separately for the participants with a DUP of 1 month or less (*n* = 52), the same trends favoring prolonged OPUS treatment were present for the negative dimension, but these results were far from significant.

**Table 2:** Outcomes 3.5-year follow-up (5 year after initiation of treatment), participants analyzed separately based on DUP, treatment delay and total treatment delay

Imputed data																		
	DUP ≤3 months						Treatment delay ≤3 months						Total treatment delay ≤6 months					
	OPUS	TAU	Estimated mean difference	*p*	Interaction analyses		OPUS	TAU	Estimated mean difference	*p*	Interaction analyses		OPUS	TAU	Estimated mean difference	*p*	Interaction analyses	
					Primary^b^	Secondary^c^					Primary^b^	Secondary^c^					Primary^b^	Secondary^c^
Psychotic dimension, mean	1.7	1.8	−0.16 (−0.8; 0.5)	0.63	0.92	0.48	1.7	2	−0.33 (−0.76; 0.11)	0.14	0.34	0.04	1.6	2.1	−0.48 (−1.3; 0.31)	0.23	0.42	0.84
Disorganized dimension, mean	0.56	0.96	−0.39 (−0.81; 0.03)	0.07	0.20	0.85	0.69	0.76	−0.07 (−0.36;0.23)	0.66	0.80	0.74	0.69	1	−0.31 (−0.82; 0.19)	0.23	0.48	0.27
Negative dimension, mean	1.5	2.0	−0.45 (−0.96; 0.05)	0.08	0.09	0.19	1.7	1.8	−0.1 (−0.46; 0.26)	0.59	0.60	0.06	1.5	2.1	−0.61 (−1.2; 0.006)	0.05	0.06	0.40
PSP, mean	54	50.9	3.1 (−3.4; 9.5)	0.35	0.31	0.75	53.5	53.4	0.13 (−4.7; 5.0)	0.96	0.50	0.13	54.8	54.2	0.58 (−8.1; 9.2)	0.90	0.85	0.79
BACS, mean	−1.9	−2.1	0.22 (−0.65;1.1)	0.63	0.63	0.54	−2.1	−2	−0.13 (−0.75; 0.48)	0.67	0.50	0.68	−1.8	−2.5	0.65 (−0.49; 1.8)	0.26	0.20	0.74
Competitive work or study per year, months, mean (SD)^a^	2.9 (4.2)	1.9 (3.2)	1.0 (−0.68; 2.7)	0.24	0.14	0.21	1.9 (3.7)	2.3 (3.8)	−0.47 (−1.6; 0.71)	0.43	0.16	0.99	2.8 (4.1)	2.7 (3.9)	0.10 (−2.1; 2.3)	0.93	0.88	0.64
Days admitted per year, mean (SD) ^a^	14 (23)	21 (28)	−7.0 (−19; 5.7)	0.24	0.48	0.75	10 (22)	16 (48)	−6.0 (−18; 5.7)	0.31	0.35	0.29	14 (24)	23 (51)	−9.3 (−31; 12)	0.39	0.37	0.49
			OR (95%)						OR (95%)						OR (95%)			
Remission, *n* (%)	9 (20)	3 (9)	2.4 (0.6; 9.7)	0.22	0.19	0.97	16 (20)	15 (19)	1.1 (0.48; 2.3)	0.87	0.71	0.06	6 (21)	1 (4)	5.7 (0.64; 51)	0.12	0.10	0.95
Diagnosis of abuse or dependency syndrome, *n* (%)	8 (18)	7 (21)	0.90 (0.26; 3.1)	0.86	0.92	0.81	13 (16)	19 (24)	0.63 (0.27; 1.5)	0.29	0.29	0.15	5 (17)	7 (28)	0.49 (0.12; 2.0)	0.32	0.29	0.36

*DUP* duration of untreated psychosis, *PSP* Personal and Social Performance scale, *BACS* Brief Assessment of Cognition in Schizophrenia, *OR* odds ratio

^a^ Data are based on registers and, therefore, there is no missing data and no need for imputation

^b^ Primary interaction analyses are for DUP and treatment delay dichotomized at 3 months, and total treatment delay at 6 months

^c^ Secondary interaction analyses are for DUP and treatment delay dichotomized at 6 months and total treatment delay at 9 months. Results beyond interactions analyses are not shown for secondary analyses

#### Treatment delay

A total of 149 participants had a treatment delay of less than 3 months. Of these, 73 were randomized to TAU and 76 to prolonged OPUS treatment. There were no differences in treatment response based on a 3-month dichotomizing of the treatment delay. If the short treatment delay was increased to 6 months (including 199 participants in the short treatment delay group), the interaction analyses indicated that the intervention treatment and the treatment delay were trend-level significant on the psychotic dimension (*p* = 0.04), on the negative dimension (*p* = 0.06), and on rates of remission (*p* = 0.06; only *p*-values of the interaction analyses are shown).

#### Total treatment delay

A total of 54 participants had a total treatment delay of <6 months; 25 of those were randomized to TAU and 29 to prolonged OPUS treatment. As shown in Table 2, there was an effect of the intervention treatment for the group with a short total treatment delay on negative symptoms (estimated mean difference −0.61, 95% CI (−1.2; 0.006), *p* = 0.05), which were lost for those participants with a total delay of more than 6 months. Increasing the total treatment delay to 9 months decreased the significance of the interactions.

Outcomes for DUP, treatment delay and total treatment delay are shown in Table 2.

#### Mode of onset

**Fig. 2:**
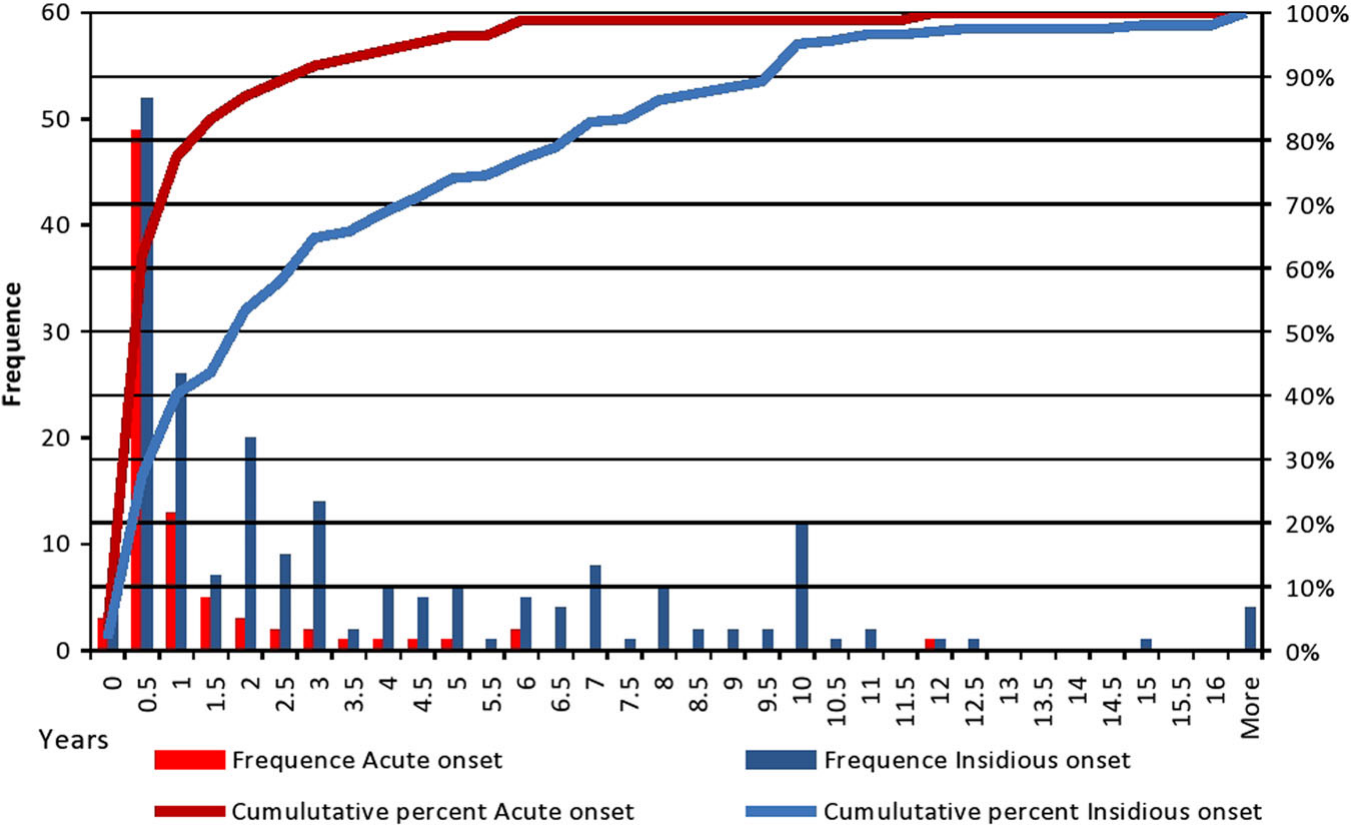
Distribution of duration of untreated psychosis in years by mode of onset of psychosis

As shown in Table 3, mode of onset was significantly associated with DUP. When the treatment response was analyzed according to mode of onset instead of DUP or treatment delay there was no evidence of a correlation between randomization and mode of onset. Distribution of DUP by mode of onset is depicted in Fig. 2.

**Table 3:** Mode of onset

	*n*	Mean DUP weeks (SD)	Median DUP weeks (range)	Mean TD weeks (SD)	Median TD weeks (range)
Acute onset (1–30 days)	84	49 (92) ^a^	12 (624)	33 (70) ^b^	11 (440)
Insidious onset (>30 days)	204	180 (201) ^a^	104 (962)	40 (72) ^b^	15 (430)
Total	288	142 (186)	52 (962)	38 (71)	14 (440)

Mann–Whitney *U*-test: *p* < 0.000 for the DUP^a^ and *p* = 0.192 for the TD^b^ (treatment delay)

## Discussion

Our findings suggest a relationship between DUP and treatment response. We found a trend effect in the intervention group on disorganized dimension and negative dimension for participants with a DUP <3 months. We did not find any beneficial effects of prolonged SEI treatment for the group with a treatment delay of <3 months; however, sensitivity analyses indicated a significant interaction on the psychotic dimension and trend for negative symptoms and remission. Participants who had a total treatment delay shorter than 6 months had a more favorable response to the intervention treatment on the negative symptoms than those with a total treatment delay of more than 6 months. Even though mode of onset was significantly associated with DUP, there was no evidence that it could explain the enhanced treatment response seen in the short DUP group.

DUP has been found to predict outcome in several studies,^10–12^ but there is no agreement on what duration should be considered long. One study divided DUP into five groups (0–7, 7–28, 28–90, 90–365, and >365 days) and found that the three shortest groups had more favorable outcomes than the longest.^19^ Another study grouped DUP at 1 month, 3 months and 1 year and found effects on the psychotic symptoms at the 8-year follow-up favoring participants with a DUP shorter than 3 months, but remission rates were higher only in the group with a DUP shorter than 1 month.^20^ A meta-analysis of the effect of DUP found significant effect on outcome, but there was no effect of what threshold the included studies had used to dichotomize between short and long DUP.^11^ We chose a relative low cutoff point in our primary analyses to ensure that most of the SEI treatment had been delivered within the 5-year critical period.

One of SEI treatment’s major achievements has been its ability to affect negative symptoms^21^ and our findings suggest that this is also true of prolonged SEI treatment provided that the treatment is provided within the first years of illness. These results are in line with the RAISE study, which found that patients with a short DUP (≤74 weeks) benefited substantially more from the 2-year intervention treatment than those with a long DUP (>74 weeks).^22^ Both these studies support the critical period hypothesis, which suggests that the later static years (plateau phase) are less receptive to treatment interventions and that the window of opportunity for changing the course of schizophrenia is within the first 5 years of illness.^23^

A recent study investigating differences in outcome based on onset of either medical treatment, SEI treatment, or both combined indicated that the beneficial impact of short DUP on outcome was strongest if the end of DUP was defined as both adequate medical treatment and enrollment in a SEI program.^24^ Our finding of an effect on the negative symptoms for participants with a total treatment delay of <6 months support the notion that adequate treatment for patients with newly diagnosed schizophrenia should be considered both medical treatment and psycho-social treatment.

We found no effect of reducing the treatment delay to 3 months, but dichotomizing the treatment delay at 6 months showed tendencies on three domains. We do not believe that this result is due to a special effect of the arbitrary 6 months dichotomizing; the finding could be due either to a type I error in the former or a type II error in the latter. Since the number of participants was increased in the secondary analyses with treatment delay dichotomized at 6 months, that these results are more likely to be closer to the true impact. This would imply that, in addition to looking into methods to shorten the DUP, we should also be aware of the importance of delays within mental health services and strive to make referral to relevant treatment facilities as prompt and effective as possible.

### Methodological considerations

This study is a sub-group analysis; the power calculations were not made to detect differences within these smaller populations. Therefore, the risk of type I and type II errors is enhanced. We, therefore, view this study as an exploratory study and caution that results should be viewed as indicative, not as definitive. However, a study such as this is important for suggesting further directions for research. Also, there is an attrition bias toward the least ill at baseline being more likely to attend the follow-up interview. We corrected for this using multiple imputations, setting the number of imputations as high as 100. At both assessment points we used raters blinded to randomization and at follow-up the raters were blinded for baseline results. Clinicians and participants were not blinded. We randomized a relatively large group; randomization procedures were carried out at an external site with investigators blinded to allocation sequence.

### Conclusion

Given the nature of the study we cannot draw any definitive conclusions on the moderating effect of DUP on the response to prolonged SEI treatment. Prior studies of SEI treatment have shown effect of treatment for shorter programs (1–3 years). The primary study^25^ of the effect of prolonged SEI treatment did not find any effect on the functional, cognitive, or psychopathological domains; however, this study finds that prolonged SEI treatment have a beneficial effect on negative symptoms, and suggests effects on other psychopathological domains, given that the treatment is provided early in the course of illness. Our findings support the critical period hypothesis in that the first years after illness onset seem to be more receptive to interventional treatment.

## Methods

### Study design

This study was a sub-group analysis of participants from a large randomized superiority trial, The OPUS II study.^25^ The main study was set up to test the effect of 5 years of SEI treatment compared to 2 years of SEI treatment followed by 3 years of standard treatment. Participants were recruited for the study on average 19 months after initiation of SEI treatment. In this particular study we aimed to test the moderating effect of DUP and treatment delay on treatment response to the prolonged SEI treatment.

### Participants

Participants were recruited from six SEI teams in Denmark, named OPUS teams, from 2009 through 2012. OPUS treatment, for the first 2 years, is the standard treatment for patients newly diagnosed with a schizophrenia spectrum disorder in Denmark (ICD 10:F20-F29).^26, 27^ The OPUS teams are part of the adult mental health services and treat patients between 18 and 35 years of age at time of admission.

At baseline assessment diagnoses were confirmed via a semi-structured interview using the Schedules for Clinical Assessment in Neuropsychiatry (SCAN).^28^ In all, 400 participants were recruited for the main trial. In this particular study, participants diagnosed with schizotypal disorder (*n* = 83) were excluded from the analyses because a diagnosis of schizotypal disorder precludes any psychotic episodes.

### Intervention treatment

The OPUS treatment has three core elements: assertive community treatment,^29^ family involvement,^30, 31^ and social skills training.^32^ The teams are multidisciplinary, consisting of psychiatrists, psychologists, psychiatric nurses, psychotherapists, and vocational therapists. The maximum patient: case manager caseload is 12:1. The treatment in the intervention period was not altered in any substantial matter; it was assumed that the proportion of patients in remission would be higher so the maximum caseload was enhanced to 15:1. The participants and the case manager should have at least two contacts per months, of which at least one should be face to face.

### Treatment as usual

Participants randomized to TAU received 2 years of OPUS treatment, after which, in most cases, they were referred to community health centers (a minority of participants were referred to their general practitioner or assertive community teams). The community health centers treat a variety of psychiatric illnesses and the caseload is approximately 26:1.

For full description of the two treatment arms, see refs. 33 and 25.

### Antipsychotic treatment

Participants, both in TAU and the intervention group, are treated in concordance with Danish national recommendations recommending low doses of second generation anti-psychotics for newly diagnosed schizophrenia spectrum disorders.^34^ Antipsychotic treatment was not a part of the randomized trial and the decision to initiate or terminate medical treatment was made by the treating clinician and the participant.

### Assessments and outcomes

Patients were interviewed at baseline and follow-up either at the research facilities or at their homes, based on their preferences. Raters were blinded to treatment allocation at both baseline and follow-up. Follow-up interviews were conducted from 2012–2015.

DUP was defined as the period from first occurrence of a psychotic symptom with an intensity equivalent to a score of 3 or above on one of two of the global domains (global hallucination and global delusion) on the Scale for Assessment of Positive Symptoms (SAPS).^35^ In line with the ÆSOP study we set the end of the DUP at the initiation of antipsychotic medication, or in the few cases where medical treatment was not initiated, at the start of specialized psychiatric treatment.^16^ In the rare cases in which participants had had a prior psychotic episode that had terminated without treatment, this period was added to the overall DUP. In accordance with the Interview for the Retrospective Assessment of the Onset of Schizophrenia (IRAOS)^36^ psychotic episodes prior to the age of 12 were not included in the DUP assessment.

In accordance with the positive skewed distribution of the DUP and prior findings that the relationship between DUP and outcome is nonlinear^37^ we ch﻿os﻿e to dichotomized DUP into short DUP (≤3 months) and long DUP (>3 months).

To investigate whether there was a delay in referrals to OPUS treatment within the mental health system, we used The Danish Psychiatric Central Research Register^38^ and identified the first time participants were registered with a psychotic diagnosis within the schizophrenia spectrum. The time period from first registered diagnosis until start of OPUS treatment was named *treatment delay;* the period represented the duration from the first time a health professional registered the diagnoses until the patient received the recommended OPUS treatment. The treatment delay, therefore, reflects the referral time to OPUS treatment. If there were waiting lists for OPUS treatment, patients were treated by the community health center while they were waiting. The treatment delay might in some cases reflect a long hospitalization or that patients were diagnosed with schizophrenia but discontinued from treatment before their referral to OPUS and at a later time started their specialized treatment. Patients who were hospitalized or treated in community health centers prior to start of their OPUS treatment would routinely be prescribed antipsychotic medication, but we have no estimate as to the extent to which this actually occurred or if patients were compliant with the treatment. Duration was dichotomized into short treatment delay (≤3 months) and long treatment delay (>3 months).

Finally, the DUP and the treatment delay were added together into one variable, Total treatment delay, and this variable was dichotomized into short total treatment delay (≤6 months) and long total treatment delay (>6 months). This variable gave an estimate of the time from first psychotic symptom until initiation of the recommended OPUS treatment.

We chose a rather short DUP as cutoff because we wanted to ensure that the prolonged 5-year treatment was provided within the first years after onset of illness. However, given the arbitrary nature of any dichotomizing, we conducted sensitivity analyses in which the DUP and treatment delay were dichotomized at 6 months and total treatment delay was dichotomized at 9 months. For the DUP, we also conducted sensitivity analyses dichotomizing the DUP around 1 month. OPUS teams are part of the adult mental health services and, therefore, patients who are diagnosed with a schizophrenia spectrum disorder prior to their 18th birthday are treated in adolescent services until their 18^th^ birthday. For sensitivity measures these participants were excluded in secondary analyses.

To assess the mode of onset of psychosis participants were asked to recollect the debut of their illness and then the investigator classified the mode of onset, in accordance with the DOSMeD study,^39^ as either acute (development of symptoms over 1 to 7 days), sub-acute (development of symptoms over 7 to 30 days) or insidious (development of symptoms over more than 30 days). For analytic purposes the patients were categorized as acute (compromising acute and sub-acute) and insidious.^16^

Psychopathological, functional, and social variables were assessed at both baseline and follow-up. DUP was assessed at the baseline interview. Investigators were blinded to randomization group and investigators at follow-up were blinded to baseline results. The groups were analyzed separately for clinical and social outcomes; psychopathology were assessed using the SAPS and Scale for Assessment of Negative Symptoms (SANS) ^35^ and calculated into a psychotic dimension, a disorganized dimension, and a negative dimension.^40^ Level of functioning was assessed using the Personal and Social Performance (PSP) scale.^41^ Cognitive level was indexed using the Brief Assessment of Cognition in Schizophrenia (BACS) ^42^ and converted into a total *z*-score using preexisting data from health controls. Employment was operationalized as number of months employed per year after randomization until follow-up and was estimated using the DREAM database^43^ provided by the Danish ministry of employment. Total number of bed days was calculated using data from the Danish Psychiatric Central Research Register.^38^

### Interrater reliability

All investigators were trained in conducting the SCAN, the SAPS, and the SANS ratings. Interrater reliability interviews were conducted during the study period. The intraclass correlation coefficient varied between 0.63 and 0.77 for the negative dimension, indicating good agreement, and between 0.7 and 0.9 on the psychotic dimension, indicating good to very good agreement.

### Randomization

Randomization was centralized and computerized with concealed randomization sequences. Block size varied from 6 to 10 and was concealed from clinicians and investigators. Participants were stratified by treatment site and level of negative symptoms. The negative symptoms were assessed on the SANS and participants were stratified by at least one score above 2 (mild symptoms) on any of the four global domains (affective flattening, alogia, avolition-apathy, and anhedonia), compared to no scores above 2.

### Statistical method

The data were analyzed using binary logistic regression for the dichotomous variables and linear regression for the continuous variables. Interactions between randomization and DUP were tested in the imputed data using linear regression for scale outcomes and binary logistic regression for the binary outcomes, using an unrestricted fraction missing information test. To compensate for missing data multiple imputations by chained equations were used (*m* = 100), linear regression was used for continuous outcomes and binary logistic regression was used for dichotomous outcomes. Age, sex, diagnosis, and the baseline values of the outcome variables were used as imputation measures.

All analyses were conducted using SPSS version 22 and Stata/SE version 13.1.

### Data availability

A full data set will be made available at the Danish National Archives (Rigsarkivet) after the initial publications. Statistical codes are available from the corresponding author at nikolai.albert@regionh.dk.
